# The effect of dolutegravir on obstetrical outcomes among women living with HIV: A retrospective cohort study

**DOI:** 10.1111/jog.16349

**Published:** 2025-06-22

**Authors:** Ammar Al Naimi, Charlotte Chang, Holly Rawizza, Oluwaseun Olaifa, Olabanjo Ogunsola, Prosper Okonkwo, Phyllis Kanki

**Affiliations:** ^1^ Department of Immunology and Infectious Diseases Harvard T. H. Chan School of Public Health Boston Massachusetts USA; ^2^ Department of Obstetrics and Gynecology Buergerhospital Frankfurt Germany; ^3^ Department of Obstetrics and Perinatal Medicine Goethe University Hospital of Frankfurt Frankfurt Germany; ^4^ Brigham & Women's Hospital Boston Massachusetts USA; ^5^ APIN Public Health Initiatives Abuja Nigeria

**Keywords:** dolutegravir, DTG, HIV, obstetrical outcomes

## Abstract

**Objective:**

To assess the effect of dolutegravir (DTG) on obstetrical outcomes for women living with HIV (WLWH).

**Methods:**

This retrospective cohort study evaluated adult WLWH in antenatal care between 2016 and 2022 at two Nigerian centers. Patients were stratified into three antiretroviral therapy (ART) exposure groups: non‐DTG ART, DTG‐switch, and DTG. Multivariate linear and logistic regression models were utilized to assess the association between the type of ART and gestational weight gain (GWG), small for gestational age (SGA), gestational hypertension, and preterm birth.

**Results:**

The study included 1839 (77.1%) non‐DTG users, 359 (15%) DTG users, and 188 (7.9%) DTG switchers. The adjusted association between DTG exposure and GWG was 0.72 (−0.08, 1.53) kg and 0.51 (−0.51, 1.54) kg for the DTG and DTG‐switch groups, respectively, compared to other ARTs. The adjusted odds ratios for gestational hypertension were 0.47 (0.09, 2.49) and 1.31 (0.43, 4.02), 0.57 (0.22, 1.52) and 0.73 (0.25, 2.10) for preterm birth, and 0.55 (0.25, 1.20) and 0.26 (0.08, 0.83) for SGA in DTG and DTG‐switch, respectively, compared to other ARTs.

**Conclusions:**

DTG therapy is not associated with the risk of gestational hypertension nor preterm birth, and antenatal ART switching to DTG decreases the risk of growth restriction.

## INTRODUCTION

Women living with HIV (WLWH) represent up to one fourth of pregnancies in countries such as Botswana due to the high prevalence of HIV in Africa; therefore, HIV‐related healthcare is an important factor affecting obstetrical outcomes of African women.^1^ Favorable neonatal outcomes with lower perinatal transmission and morbidity are associated with early antenatal initiation of antiretroviral therapy (ART).^2^

Dolutegravir (DTG), a second‐generation integrase strand transfer inhibitor (INSTI), was FDA approved in 2013 and is currently a component of preferred ART regimens globally due to low cost, convenient dosing, few drug–drug interactions, and a high genetic barrier to resistance.^3^ DTG showed superior viral suppression during pregnancy without increasing the risk of preterm birth or growth restriction.^4^ The DolPHIN‐2 trial proved the safety and efficacy of DTG during pregnancy with maintained postpartum low viral load; thus, it became the recommended ART with a suitable dual NRTI backbone for women of childbearing age.^5^, ^6^, ^7^

Nevertheless, several studies, including the ADVANCE and NAMSAL trials, showed an association between DTG and clinically significant weight gain in adults.^8^, ^9^, ^10^ This increase in weight could be attributed to the proinflammatory effect of DTG on neutrophils.^11^ Nevertheless, multiple factors could lead to this weight gain including reduction of inflammatory catabolism, lower energy expenditure, or changes in habits and behavior.^8^ Excessive maternal weight gain during pregnancy is associated with gestational diabetes, gestational hypertension, preeclampsia, macrosomia, preterm birth, obstructed labor and other adverse obstetrical outcomes.^12^, ^13^, ^14^, ^15^ Published data about the weight‐related outcomes of WLWH who receive DTG treatment during pregnancy are scarce and contradictory.

The APIN‐PEPFAR Program supports HIV care and treatment services for adults and children, including pregnant WLWH, at numerous clinics and hospitals in Nigeria; for this research study, we examined data from two large antenatal clinics in north‐central Plateau and Benue state.^16^

The aim of this study was to assess the effect of DTG as well as maternal weight gain during pregnancy, referred to as gestational weight gain in this manuscript, on the obstetrical outcomes for WLWH.

## METHODS

In this retrospective cohort study, we utilized data that was collected for routine clinical care in the APIN PEPFAR HIV clinical program in Nigeria with a national HIV prevalence of 1.3%.^17^ Adult pregnant WLWH entering the antenatal care program for a new pregnancy between January 1, 2016 and June 30, 2022 at Jos University Teaching Hospital (JUTH) and Federal Medical Centre Makurdi (FMCM), who provided written informed consent allowing use of their data in secondary research, were identified.

The electronic clinical records of their enrollment assessment, adult care visits, antenatal care visits, delivery data, laboratory results, and pharmacy visits were acquired and linked. Women infected with only HIV‐2 and women younger than 18 years at the earliest point of follow‐up time were excluded.

WLWH were classified into three ART exposure groups based on pharmacy records: the DTG group was receiving DTG prior to the start of pregnancy, the DTG‐switch group started pregnancy on any different ART and switched to DTG prior to delivery, and the non‐DTG group did not receive DTG during the entire course of gestation.

Our obstetrical outcomes included two continuous outcomes (net gestational weight gain, mean neonatal birth weight) and two binomial outcomes (gestational hypertension, preterm birth prior to completed 37 gestational weeks). Multivariate linear and logistic regression models assessed the association between DTG exposure and the continuous and binomial outcomes, respectively. Combinations of covariates, including the center of care, weight at baseline prior to pregnancy, maternal age, systolic blood pressure, diastolic blood pressure, timing of first antenatal visit, gestational age at delivery, education, and occupation, were accounted for as potential confounders in the adjusted analysis.

Sensitivity analysis for the outcome of neonatal birth weight was performed by dichotomizing newborns into small for gestational age (SGA) versus normal and utilizing logistic regression to assess the association of ART with the risk of SGA using the same covariates of the linear regression model for continuous neonatal birth weight. Other sensitivity analyses involved the transformation of the DTG exposure into a time‐varying exposure by including a dichotomous exposure variable, initiation time, and an interaction term instead of the stratified categorical exposure.

All statistical analyses were conducted with Stata® (ver. 18, Texas, USA), and the cut off point for statistical significance was a *p*‐value of 0.05. This study was approved by the APIN Institutional Review Board (ref: IRB‐SD) and the Institutional Review Board of the Harvard T.H. Chan School of Public Health (ref: IRB23‐0687).

## RESULTS

A total of 2386 women were included in this study, of whom 1839 (77.1%) were non‐DTG users, 359 (15%) were DTG users throughout the entire pregnancy, and 188 (7.9%) switched to DTG during gestation at about 4.7 ± 2.8 gestational months.

The mean maternal age was 32.8, 33.9, and 33.7 years for the non‐DTG users, DTG switchers, and DTG users, respectively. The DTG users were more prevalent at FMCM than JUTH (66.6% vs. 33.43%), had lower baseline RNA viral load (3904/mL), higher CD4 count (577 cells/μL), and had their first ANC visit slightly earlier (19.8 weeks) than non‐DTG users and the DTG switchers. The demographic data of the three cohorts is summarized in Table 1.

**TABLE 1:** Summary of demographic data by DTG exposure, with frequency of missing observations for each variable.

Demographic data (*N* = 2386), missing data	Non‐DTG *N* = 1839	Switch to DTG *N* = 188	DTG *N* = 359
Maternal age (years), 0	32.8 (5.1)	33.9 (4.9)	33.7 (4.8)
History of hypertension, 188	20 (1.2%)	1 (0.6%)	4 (1.2%)
History of diabetes, 190	4 (0.2%)	0 (0.0%)	1 (0.3%)
Center, 0			
JUTH	1033 (56.2%)	103 (54.8%)	120 (33.4%)
FMCM	806 (43.8%)	85 (45.2%)	239 (66.6%)
Education, 64			
None	201 (11.2%)	14 (7.5%)	36 (10.3%)
Primary	398 (22.3%)	39 (21.0%)	60 (17.2%)
Secondary	718 (40.2%)	82 (44.1%)	166 (47.7%)
Tertiary	471 (26.3%)	51 (27.4%)	86 (24.7%)
Occupation, 69			
Clerical support workers	106 (6.0%)	8 (4.3%)	14 (4.0%)
Services and sales workers	825 (46.3%)	86 (46.5%)	165 (47.0%)
Professionals	114 (6.4%)	11 (5.9%)	26 (7.4%)
Students	246 (13.8%)	24 (13.0%)	52 (14.8%)
Housewives	269 (15.1%)	36 (19.5%)	53 (15.1%)
Unemployed	221 (12.4%)	20 (10.8%)	41 (11.7%)
Married, 85	1091 (61.8%)	118 (63.8%)	228 (64.8%)
History of tuberculosis, 78	40 (2.3%)	3 (1.6%)	13 (3.7%)
Risk for preterm birth, 1104	168 (17.1%)	25 (23.1%)	41 (21.1%)
RNA viral load, 176	13 071 (83264)	6363 (33062)	3904 (40842)
CD4 count, 599	513 (227)	531 (254)	577 (250)
Hemoglobin, 1243	12.3 (3.6)	12.1 (1.2)	12.4 (1.0)
ART regimens at baseline, 0			
Dolutegravir	0 (0%)	0 (0%)	359 (100%)
Efavirenz	534 (29.0%)	40 (21.3%)	0 (0%)
Nevirapine	404 (22.0%)	58 (30.8%)	0 (0%)
Lopinavir/ritonavir	40 (2.2%)	2 (1.1%)	0 (0%)
Other	861 (46.8%)	88 (46.8%)	0 (0%)
First antenatal care visit (gestational weeks), 220	22.7 (7.4)	22.1 (8.2)	19.8 (8.3)
Weight at baseline (kg), 22	64.0 (13.7)	65.3 (12.7)	63.0 (11.9)
BMI at baseline (kg/m^2^), 1685	31.0 (86.8)	25.7 (5.5)	25.5 (6.2)

*Note*: Data presented as mean (standard deviation) or frequency (proportion, %).

Abbreviations: ART, antiretroviral therapy; BMI, body mass index; DTG, dolutegravir; FMCM, Federal Medical Centre Makurdi; JUTH, Jos University Teaching Hospital.

The mean gestational age at delivery was 38.7 ± 2.0 weeks, and neonatal birth weight was 3.1 ± 0.7 kg. Most deliveries were vaginal 1350 (80.9%) and the frequency of female neonates was 930 (51.4%). The risk of miscarriage was 1.5% and 121 (5.1%) women developed gestational hypertension. The DTG‐switch group had higher mean neonatal birth weight 3.4 ± 0.8 kg, a larger neonatal head circumference 36.1 ± 1.9 cm, and a higher likelihood of cesarean delivery 24.6%. The obstetrical outcomes stratified by the DTG exposure groups are summarized in Table 2.

**TABLE 2:** Summary of obstetrical outcomes stratified by antenatal ART exposure groups.

Obstetrical outcomes	Non‐DTG *N* = 1839	Switch to DTG *N* = 188	DTG *N* = 359	*p*‐Value
History of preterm	168 (17.1%)	25 (23.1%)	41 (21.1%)	0.16
Neonatal birth weight	3.0 (0.7)	3.4 (0.8)	3.1 (0.6)	**<0.001**
Neonatal length	48.8 (4.0)	49.9 (3.7)	49.1 (3.8)	0.11
Neonatal head circumference	35.3 (2.1)	36.1 (1.9)	35.3 (1.9)	**0.016**
Gestational age at delivery	38.7 (1.9)	38.8 (1.4)	38.8 (2.5)	0.64
Apgar score	8.0 (8.0–8.0)	8.0 (8.0–8.0)	8.0 (8.0–8.0)	0.95
Delivery				
Normal vaginal	1078 (80.6%)	97 (74.6%)	168 (83.2%)	**0.01**
Assisted vaginal	6 (0.4%)	1 (0.8%)	0 (0.0%)	
Elective cesarean	153 (11.4%)	26 (20.0%)	14 (6.9%)	
Emergency cesarean	100 (7.5%)	6 (4.6%)	20 (9.9%)	
Infant female sex	743 (51.5%)	69 (50.0%)	118 (52.0%)	0.93
Miscarriage	13 (1.5%)	1 (1.1%)	1 (1.3%)	0.95
Feeding				
Breast feeding	824 (92.3%)	80 (89.9%)	74 (94.9%)	0.77
Breastmilk substitute	63 (7.1%)	8 (9.0%)	4 (5.1%)	
Mixed	6 (0.7%)	1 (1.1%)	0 (0.0%)	
Gestational hypertension	98 (5.3%)	13 (6.9%)	10 (2.8%)	0.065
Mean gestational weight gain	4.1 (6.7)	4.2 (7.0)	4.8 (6.9)	0.083

*Note*: Data presented as mean (standard deviation) or frequency (proportion, %).

Abbreviations: ART, antiretroviral therapy; DTG, dolutegravir.

The mean gestational weight gain was 4.2 ± 6.8 kg and a linear regression model for the association between DTG exposure and the net gestational weight gain in kg after adjusting for eight covariates resulted in β coefficients of 0.72 (−0.08, 1.53) and 0.51 (−0.51, 1.54) for the DTG and DTG‐switch groups, respectively, compared to other ARTs (Table 3). This indicates no significant differences in gestational weight gain between the groups.

**TABLE 3:** The linear regression model for the association between ART and net gestational weight gain adjusting for center of care, weight at baseline, maternal age, systolic blood pressure, diastolic blood pressure, education, occupation, and timing of the first antenatal visit (*n* = 2056).

Outcome: gestational weight gain (kg)	β coefficient (0.95 CI)	*p*‐Value
ART (reference non‐DTG)		
Switch to DTG	0.51 (−0.51, 1.54)	0.33
DTG	0.72 (−0.08, 1.53)	0.08
Center of care (reference JUTH)	−0.36 (−0.96, 0.23)	0.23
Baseline weight (kg)	−0.15 (−0.17, −0.12)	**<0.001**
Maternal age (years)	−0.018 (−0.08, 0.04)	0.55
Systolic blood pressure (mmHg)	0.0002 (−0.00003, 0.0004)	0.09
Diastolic blood pressure (mmHg)	0.004 (−0.013, 0.022)	0.63
Education (reference none)		
Primary	0.20 (−0.86, 1.26)	0.72
Secondary	0.29 (−0.69, 1.27)	0.56
Tertiary	1.11 (−0.02, 2.25)	0.06
Occupation (reference clerical support workers)		
Services and sales workers	−0.14 (−1.44, 1.15)	0.83
Professionals	0.24 (−1.33, 1.81)	0.76
Students	0.34 (−1.05, 1.72)	0.63
Housewives	0.52 (−0.89, 1.94)	0.47
Unemployed	−0.25 (−1.68, 1.18)	0.73
First antenatal visit (weeks)	0.02 (−0.013, 0.06)	0.20
Intercept	12.78 (9.70, 15.85)	<0.001

Abbreviations: ART, antiretroviral therapy; CI, confidence interval; DTG, dolutegravir; JUTH, Jos University Teaching Hospital.

The mean neonatal birth weight was 3.06 ± 0.69 kg. A linear regression model for the association between antenatal DTG exposure and the mean birth weight in kg after adjusting for seven covariates resulted in β coefficients of 0.10 (−0.05, 0.24) and 0.35 (0.20, 0.50) for the DTG and DTG‐switch groups, respectively, compared to other ARTs. This indicates an increase of birth weight by 0.35 (0.20, 0.50) kg for the DTG‐switch group compared to other ARTs (Table 4).

**TABLE 4:** The linear regression model for the association between neonatal birth weight and ART, adjusting for net gestational weight gain, center of care, weight at baseline, maternal age, education, occupation, first antenatal visit, and gestational age at delivery (*n* = 994).

Outcome: birth weight (kg)	β coefficient (0.95 CI)	*p*‐Value
ART (reference non‐DTG)		
Switch to DTG	0.35 (0.20, 0.50)	**<0.001**
DTG	0.10 (−0.05, 0.24)	0.18
Maternal gestational weight gain (kg)	0.01 (0.01, 0.02)	**<0.001**
Center of care (reference JUTH)	−0.21 (−0.32, −0.11)	**<0.001**
Baseline weight (kg)	0.01 (0.01, 0.01)	**<0.001**
Maternal age (years)	−0.01 (−0.02, −0.00002)	**0.05**
Education (reference none)		
Primary	−0.005 (−0.18, 0.17)	0.96
Secondary	0.02 (−0.15, 0.18)	0.86
Tertiary	0.05 (−0.13, 0.23)	0.61
Occupation (reference clerical support workers)		
Services and sales workers	−0.08 (−0.27, 0.10)	0.37
Professionals	−0.04 (−0.27, 0.19)	0.72
Students	−0.04 (−0.24, 0.16)	0.72
Housewives	−0.004 (−0.21, 0.20)	0.97
Unemployed	0.06 (−0.15, 0.26)	0.57
First antenatal visit (weeks)	−0.005 (−0.01, 0.001)	0.13
Gestational age at delivery (weeks)	0.04 (0.01, 0.06)	**0.001**
Intercept	1.46 (0.52, 2.40)	0.002

Abbreviations: ART, antiretroviral therapy; CI, confidence interval; DTG, dolutegravir; JUTH, Jos University Teaching Hospital.

Gestational hypertension developed in 121 (5.1%) patients. A multivariate logistic regression model for the association between the risk of gestational hypertension, ART, while adjusting for 10 covariates showed no effect of DTG on gestational hypertension and an odds ratio of 1.08 (1.02, 1.14) indicating an 8% increase in the odds of gestational hypertension for each kilogram of gestational weight gained independent of the ART group (Table 5).

**TABLE 5:** The logistic regression model for the association between gestational hypertension and ART, adjusting for gestational weight gain, center of care, weight at baseline, maternal age, systolic blood pressure, diastolic blood pressure, education, occupation, first antenatal visit, and gestational age at delivery (*n* = 1147).

Outcome: gestational hypertension	Odds ratio (0.95 CI)	*p*‐Value
ART (reference non‐DTG)		
Switch to DTG	1.31 (0.43, 4.02)	0.63
DTG	0.47 (0.09, 2.49)	0.38
Maternal gestational weight gain (kg)	1.08 (1.02, 1.14)	**0.009**
Center of care (reference JUTH)	0.50 (0.14, 1.84)	0.30
Baseline weight (kg)	1.03 (1.005, 1.06)	**0.02**
Maternal age (years)	1.08 (0.99, 1.18)	0.09
Systolic blood pressure (mmHg)	1.08 (1.04, 1.12)	**<0.001**
Diastolic blood pressure (mmHg)	1.11 (1.06, 1.17)	**<0.001**
Education (reference none)		
Primary	0.67 (0.17, 2.68)	0.57
Secondary	0.63 (0.18, 2.20)	0.47
Tertiary	0.65 (0.16, 2.65)	0.55
Occupation (reference clerical support workers)		
Services and sales workers	1.10 (0.29, 4.22)	0.89
Professionals	0.14 (0.01, 1.70)	0.12
Students	0.67 (0.14, 3.18)	0.62
Housewives	1.54 (0.33, 7.09)	0.58
Unemployed	1.28 (0.26, 6.01)	0.79
First antenatal visit (weeks)	0.99 (0.94, 1.04)	0.72
Gestational age at delivery (weeks)	0.94 (0.81, 1.09)	0.40
Intercept	2.61e−10 (5.80e−14, 1.18e−06)	<0.001

Abbreviations: ART, antiretroviral therapy; CI, confidence interval; DTG, dolutegravir; JUTH, Jos University Teaching Hospital.

Seventy‐two (5.4%) newborns were delivered prematurely, and a logistic regression model for the association between antenatal DTG exposure and premature birth as an outcome after adjusting for net gestational weight gain, center of care, weight at baseline, maternal age, education, occupation, and first antenatal visit resulted in an odds ratio of 0.57 (0.22, 1.52) and 0.73 (0.25, 2.10) with *p*‐values 0.27 and 0.56 for the DTG and DTG‐switch groups respectively compared to other ARTs.

Classifying neonatal birth weight into small for gestational age (SGA) or not resulted in 144 (11.1%) SGA newborns, and a logistic regression model for the association between antenatal DTG exposure and SGA as an outcome after adjusting for gestational weight gain, center of care, gestational age at delivery, weight at baseline, maternal age, education, and occupation resulted in odds ratio of 0.55 (0.25, 1.20) and 0.26 (0.08, 0.83) with *p*‐values 0.13 and 0.02 for the DTG and DTG‐switch groups respectively compared to other ARTs. Therefore, this sensitivity analysis confirmed the association between switching to DTG and increased maternal gestational weight. All other sensitivity analyses for the obstetrical outcomes with linear and logistic regression models using the exposure to DTG during the antenatal period as a binomial variable and including DTG initiation time and an interaction term did not alter the *p*‐values of DTG exposure across the threshold of significance.

## DISCUSSION

Despite its approval and recommendation as a first‐line treatment for WLWH during pregnancy, ongoing surveillance of obstetrical outcomes for women on DTG during pregnancy is recommended due to concerns about the safety of first trimester DTG exposure.^18^ Additionally, there is a well‐established association between gestational weight gain and obstetrical outcomes. Excessive gestational weight gain is linked to the risk of preeclampsia and gestational hypertension, whereas inadequate maternal weight gain is associated with premature labor and fetal growth restriction leading to SGA.^13^, ^19^, ^20^

Published literature is inconsistent regarding the association of DTG with preterm birth, gestational hypertension, and SGA. Predictive modeling of a hypothetical sample based on the ADVACE trial showed that ART‐associated maternal weight gain and obesity increase the risk of preterm birth and gestational hypertension by 33% and 360%, respectively, while the risk of SGA is decreased by 15%.^21^ The IMPAACT randomized trial demonstrated that WLWH initiating ART in pregnancy had insufficient gestational weight gain. Women on DTG did show greater gestational weight, and this weight reduced the hazards from insufficient weight gain, including preterm labor and SGA.^22^ Our data show that DTG compared to other ARTs does not increase the risk of gestational hypertension once gestational weight gain is adjusted for. Nevertheless, higher gestational weight gain was associated with an increased risk of gestational hypertension. We hypothesize that a larger sample size of women on DTG prior to pregnancy could have led to a statistically significant higher gestational weight gain and, therefore, increased the risk of gestational hypertension. Other longer follow‐up single‐center studies observed an association between DTG and preeclampsia with OR around 4.7.^18^

Patel et al. showed that the risk of SGA, preterm birth (<37 week), and lower birth weight was slightly higher for DTG therapy compared to other ARTs, but not to the extent of being statistically significant. Their data indicated that an association between the timing of ART initiation and the obstetrical outcomes might depend on the type of ART.^4^ A recent meta‐analysis showed no evidence for the association between antenatal INSTI exposure and adverse perinatal outcomes including preterm labor and SGA.^23^ DTG exposure in our cohort did not increase the risk for preterm labor, and it showed a protective effect against SGA. Neonates of DTG‐switchers had higher birth weight, and DTG seems to have a negative association with SGA. Transplacental DTG transfer is high with an expected fetal‐maternal ratio of 34%–60%. The expected DTG median elimination half‐life in newborns is more than 30 h.^24^ Therefore, neonates could exhibit DTG‐associated weight gain rapidly and subsequently have a lower risk of SGA. DTG is the recommended regimen for ART‐naïve pregnant WLWH due to its efficacy in rapidly decreasing the viral load, and our finding of a protective effect against SGA would further support this recommendation.^7^

ART‐switching to an INSTI leads to weight gain in adults and an increase in body mass index within 8 months of a switch.^25^ Therefore it is interesting that our study did not show significant differences in gained gestational weight among the treatment groups. This could be caused by a relatively high physiological gestational weight gain compared to the DTG‐associated weight gain.

The strengths of this work lie in the large number of subjects, lengthy longitudinal data, adjusted statistical models with sensitivity analyses, and increased generalizability due to the nature of collected data. Nevertheless, limitations inherent to a retrospective design can be considered study weaknesses. Our data source did not include information on race or ethnicity of the cohort to be included as a covariate; nevertheless, it is reasonable to assume that the study population is indigenous to Nigeria. The inclusion of socioeconomical status indicators such as occupation and education, though, is a strength that allowed for including these important covariates in the multivariate regression analyses. Information bias could affect any study design and erode the validity of conclusions. Moreover, we were unable to report the incidence of congenital malformations in our cohort due to the lack of this information from the database. However, the prior concerns about the risk of neural tube defects with DTG have already been shown to be unsubstantiated.^26^ Furthermore, our study sample size would not be adequately powered to detect significant differences in rare outcomes like birth defects. One further weakness of our study is the lack of information regarding maternal‐fetal transmission rates. Neonatal data after delivery were censored and, therefore, we were unable to identify and report the effect of different ARTs on maternal‐fetal transmission. Our neonatal outcomes suffered from attrition of observations due to missing data on birth outcomes. Our rate of missing data is similar to those found by a previous study showing a retention of WLWH in Nigeria during antenatal care of about 66%.^27^

In conclusion, our data are supportive of DTG‐based ART for WLWH during pregnancy, which aligns with the World Health Organization recommendation of DTG‐based ART as unconditional first‐line and second‐line therapy for all populations.^28^ DTG therapy is not associated with the risk of gestational hypertension or preterm birth, and antenatal ART switching to DTG could be beneficial for neonates by decreasing the risk of growth restriction.

## AUTHOR CONTRIBUTIONS

**Ammar Al Naimi:** Conceptualization; formal analysis; writing – review and editing; writing – original draft; methodology. **Charlotte Chang:** Conceptualization; writing – review and editing; software; validation. **Holly Rawizza:** Conceptualization; writing – review and editing. **Oluwaseun Olaifa:** Data curation; writing – review and editing. **Olabanjo Ogunsola:** Writing – review and editing; data curation. **Prosper Okonkwo:** Writing – review and editing; data curation. **Phyllis Kanki:** Conceptualization; supervision; project administration; writing – review and editing; methodology.

## CONFLICT OF INTEREST STATEMENT

All other authors declare no competing interests.

## Data Availability

Ownership of the underlying study data belongs to the APIN Public Health Initiatives (Abuja, Nigeria), which has legal and ethical restrictions in place that prevent the public sharing of patient‐level clinical data. However, to ensure long‐term data storage and availability, data requests from interested researchers can be made to the APIN Public Health Initiatives Data Management Committee (dmc@apin.org.ng), which stores and manages access to the cleaned dataset and data dictionary used for the analyses.
